# Effects of Daehwang-Hwanglyoun-Sasim-Tang on brain injury and cognitive function in mice caused by bilateral common carotid artery stenosis

**DOI:** 10.7150/ijms.77879

**Published:** 2022-10-31

**Authors:** Myeong-Hwa Lee, Chiyeon Lim, Sehyun Lim, Suin Cho, Kyung-Min Kim

**Affiliations:** 1 College of Korean Medicine, Dong-Eui University, Busan 47227, Republic of Korea.; 2 Department of Radiology, Massachusetts General Hospital and Harvard Medical School, Massachusetts 02129, USA.; 3 College of Medicine, Dongguk University, Goyang 10326, Republic of Korea.; 4 School of Public Health, Far East University, Eumseong, 27601, Republic of Korea.; 5 School of Korean Medicine, Pusan National University, Yangsan 50612, Republic of Korea.

**Keywords:** Traditional medicine, herbal medicine, decoction, vascular dementia

## Abstract

Among geriatric diseases, cerebrovascular disease ranks fourth according to the Causes of Death Statistics in 2019, Korea, and is the most common cause of acquired disorders in adults. Daehwang-Hwanglyoun-Sasim-Tang (DHST), a herbal prescription consisting of two herbal medicines, Rhei Rhizoma and Coptidis Rhizoma, has been reported to have anti-inflammatory, antioxidant, and anticancer effects. This study was conducted to confirm the anti-inflammatory mechanism of DHST treatment in ischemic brain injury and to confirm the role of DHST in cognitive function improvement. C57BL/6 male mice were randomly divided into four groups (sham operation, bilateral common carotid artery stenosis (BCAS) control, experimental group administered 5 mL/kg DHST, experimental group administered 50 mL/kg DHST), with each group containing five mice. After 1 week, DHST was orally administered for 4 weeks, 5 days a week, and then behavioral evaluation of learning and memory was performed. In addition, morphological changes in the neurons in the CA1 region of the hippocampus were observed. Inflammation-related factors were evaluated using western blot analysis. In the 50 mL/kg DHST (H-DHST) group, the expression of apoptosis-related proteins was reduced and neuronal damage was suppressed in the hippocampal CA1 region. However, cognitive improvement was observed in the H-DHST group that was attributable to anti-inflammatory and antiapoptotic pathways. In the 5 mL/kg DHST group, no significant effect was observed compared with the control group.

## Introduction

According to the Causes of Death Statistics in 2019, Korea, cerebrovascular disease ranks fourth after cancer, heart disease, and pneumonia, and its prevalence rapidly increases after the age of 50 1. Cerebrovascular disease causes neurological damage in >60% of patients who survive, with impairments in cognitive function, hemiplegia, dysarthria, dysphagia, and depression. Moreover, it is the most common cause of acquired disorders in adults 2,3.

Alzheimer's disease is the most representative cause of dementia that accounts for 60%-70% of all dementias and is classified as degenerative and irreversible dementia. The next most common form is vascular dementia (VD), a syndrome caused by cerebrovascular disease, which accounts for ~30% of all dementias and is classified as preventable dementia 2-4. Alzheimer's disease and VD in common abnormally decrease cerebral blood flow, inhibit mitochondrial function and protein production and cause an imbalance between the production and removal of reactive oxygen species (ROS). The induction of oxidative damage in vascular endothelial, glial, and nerve cells is the main pathological mechanism for cognitive impairment 5-7.

Herbal medicines, such as Ginkgonis Flos 8, Polygalae Radix 9,10, and Acori Graminei Rhizoma 11,12, have been reported to be effective in improving learning and memory impairments that occur due to low cerebral blood flow. Moreover, Polygalae Radix and Acori Graminei Rhizoma improve learning and treat memory disorders by inhibiting the death of hippocampal neurons 9-12.

Daehwang-Hwanglyoun-Sasim-Tang (DHST) is a herbal prescription that consists of two herbal medicines, Rhei Rhizoma and Coptidis Rhizoma. Because interest in traditional medicine has increased, research on Rhei Rhizoma, Coptidis Rhizoma, and DHST has also increased. DHST was reported to be effective in the recoverability of ICR mouse from stressful conditions 13, suppressing fat accumulation in HepG2 cells 14, and improving symptoms in patients with cerebral infarction accompanied by chest pain, insomnia, and constipation 15.

Rhei Rhizoma was reported to have antithrombotic properties 16, improve lipid status 17, and reduce arterial blood pressure 18. In addition, Rhei Rhizoma was reported to inhibit vascular inflammation and atherosclerosis 19 and have an antioxidant and anti-inflammatory effect on reflux esophagitis in rats 20. Studies on cerebrovascular disease have verified the neuroprotective effect of Rhei Rhizoma in the early stages of cerebral ischemic injury 21 and the preventive and therapeutic effect on Alzheimer's disease 22. Rhei Rhizoma contains sennoside A, an active anthraquinone glucoside and anthraquinones, such as aloe-emodin, rhein, emodin, chrysophanol, and physcion 13,16.

Studies on the effect of Coptidis Rhizoma in suppressing inflammation 20,23, inhibiting ROS production 23, and maintaining the mitochondrial membrane potential 24 have been reported. Consistent with the effects of Rhei Rhizoma, Coptidis Rhizoma suppressed inflammation. In one study, the alkaloids extracted from Coptidis Rhizoma were reported to play an important neuroprotective role against cerebral ischemia/reperfusion injury in rats 25. Coptidis Rhizoma contains protoberberine-type alkaloids, such as berberine, palmatine, and coptisine 13,25.

In Asian traditional medicine, Rhei Rhizoma and Coptidis Rhizoma have frequently been used together 13-15. Both have anti-inflammatory and antioxidant effects 20 and are used against hypertension, hyperlipidemia, and cardiovascular and cerebrovascular diseases 26-28. Thus, Coptidis Rhizoma is expected to exert a significant effect on VD and cognitive impairment; however, data on the effect of Coptidis Rhizoma and the mechanisms involved are lacking.

Experiments inducing cerebral ischemia through the occlusion of cerebral blood vessels in rats have the advantages of good recovery, low mortality rate, easy behavioral experiments, and verification of drug efficacy in a short period of time and are widely used in brain disease research 29. Until recently, the bilateral common carotid artery occlusion (BCAO) model was common as an experimental animal model for research on VD 30. Among rodents, rats quickly adapt to BCAO through collateral flow owing to the anastomosis between the carotid and vertebral arteries. Therefore, the effect of carotid occlusion cannot be confirmed. In mice, it depends on the strain; however, in some species, the posterior communicating artery does not develop, resulting in extensive infarction in carotid artery occlusion, which can lead to the death of the animal 31. In the bilateral common carotid artery stenosis (BCAS) model used in this study, microcoils were overlaid on both common carotid arteries to induce stenosis. Cerebral blood flow (CBF) temporarily decreased by 60%-70% compared with the control group; however, after 1-3 months, CBF was restored to ~80% and maintained 29-31. To proceed with a pathophysiologically similar experiment, we performed this study using the BCAS model.

In this study, DHST was orally administered 1 week after BCAS surgery and continued for 4 weeks in mice with chronic cerebrovascular hypoperfusion induced by BCAS to observe the effect of DHST on VD. The Y-maze test and the novel object recognition test (NORT) were then used to measure learning and memory behavior. In addition, western blot analysis was used for evaluating protein expression.

## Materials and Methods

### Experimental animals

Six-week-old male C57BL/6 mice (20-22 g) were obtained from Samtako Bio (Osan, Korea). They were housed in a temperature and humidity-controlled polypropylene cage at 24 ± 4 °C under a light-dark (12 h-12 h) cycle, fed with a standard pellet diet, and provided water *ad libitum* for at least 1 week prior to the experiment. All experiments were approved by the Ethics Committee for Animal Care of Pusan National University (Approval No. PNU 2019-2486), which is certified by the Korean Association of Laboratory Animal Care.

### Reagents

Saline was from JW Pharmaceutical Co., Ltd. (Seoul, Korea); methanol from SK chemicals (Ulsan, Korea); cresyl violet (CV) and 2,3,5-triphenyl-tetrazolium chloride from Sigma-Aldrich Co. (St. Louis, MO, USA); and phosphate-buffered saline (PBS) from Bio Basic Inc. (Markham, Ontario, Canada). The optimal cutting temperature (OCT) compound cryostat embedding medium was purchased from Thermo Fisher Scientific (Waltham, MA, USA). The primary antibodies against c-Jun N-terminal kinase (JNK), phospho-JNK (p-JNK), protein kinase B (Akt), p-Akt, manganese superoxide dismutase (Mn-SOD), and β-actin were purchased from Cell Signaling Technology (Danvers, MA, USA). The secondary antibodies, goat anti-rabbit IgG pAb and goat anti-mouse IgG pAb, were obtained from Enzo Life Sciences Inc. (Farmingdale, NY, USA). Protein extraction solution was purchased from iNtRON (Seongnam-si, Gyeonggi-do, Korea). The BCA reagent, bovine serum albumin (BSA) standard, and enhanced chemiluminescence (ECL) western blotting chemiluminescent substrate were from Thermo Fisher Scientific (Waltham, MA, USA).

### Preparation of DHST extracts

Modified DHST was composed of two herbs (Table 1). Coptidis Rhizoma was obtained from Gwangmyung Natural Pharmaceuticals (Ulsan, Korea) and Rhei Rhizoma from Hwanin Natural Pharmaceuticals (Busan, Korea). The herbs were prepared in proportions ten times their weight for the DHST decoction. DHST was extracted by decocting Coptidis Rhizoma with boiling distilled water (500 mL) for 90 min and then adding Rhei Rhizoma for an additional 30 min. Based on the metabolic rate of mice, 50 mL/kg DHST was orally administered. This is equivalent to a daily dose of a 60 kg adult. To validate the quality of Rhei Rhizoma and Coptidis Rhizoma, high-performance liquid chromatography (HPLC) analysis of major compounds was performed (supplementary Figure 1, Figure S1).

### Preparation of the BCAS-induced VD model

BCAS procedures were performed to induce the VD mouse model. In brief, the mice were anesthetized using isoflurane in N_2_O/O_2_ (70%/30%) gas until they showed no response to mechanical stimuli to the tail. A midline cervical incision was made, and both isolated common carotid arteries (CCA) were wrapped with a microcoil (inner diameter of 0.18 mm; Sawane Spring Co., Hamamatsu, Japan) to induce CCA stenosis. The body temperature of the mice was maintained at 37 ± 0.5 °C with a heating pad.

### Treatment with DHST

Experimental mice were randomly divided into four experimental groups (n = 5): (1) sham-operated (sham), (2) BCAS + equal volumes of vehicle (BCAS control group), (3) BCAS + low-dose DHST (L-DHST), and (4) BCAS + high-dose DHST (H-DHST). The sham group consisted of mice that had surgery but not BCAS. L-DHST and H-DHST were treated with 5 and 50 mL/kg DHST, respectively. Oral gavage treatment was started 1 week after BCAS induction and continued for 4 weeks, five times a week. Figure S2 shows the schematic procedure of the experiment.

### Test for spontaneous alternation behavior

Working memory function and exploration behavior were evaluated using the spontaneous alternation test in a single session of Y-maze 32, which consists of three equal arms (35 cm length × 7 cm width × 40 cm height per arm) placed at equivalent angles and a central area (Figure S2A). Each mouse was placed at the center of the maze and allowed to explore freely for 8 min after a habituation phase of 2 min in one arm of the maze. In each test, spontaneous alternations were recorded visually by a person blinded to the experiment. Arm entry was scored when a mouse placed four paws within an arm. Spontaneous alternation was determined when entry into the three arms occurred on consecutive choices in triplet sets (e.g., C-A-B, B-C-A, and A-B-C). Spontaneous alternation behavior was calculated using the following equation: percent alternation = ([number of alternations] / [total number of arm entries - 2]) × 100. When spontaneous alternation rates increase and subjects try to explore new objects, working memory is considered to have improved.

### NORT evaluation

This test is used to assess the neophiliac tendency of rodents toward novel objects compared with familiar objects 33,34. In the acclimatization phase, each mouse was allowed to explore the open-field arena (40 × 40 × 40 cm (height) gray box) for 5 min without the objects (Figure S3B). In the first trial, two identical objects (red rounds in Figure S3B) were placed in two opposite corners of the testing arena, and the animal was allowed to explore the two objects for 10 min, during which the amount of time the animal explored was scored. After 20 min, in the second trial, the mouse was placed in the arena again, and one of the identical objects (familiar (F)) supplied in the first trial was replaced with a new identical object (new (N)). The amount of time spent exploring each object at a distance of less than 2 cm was recorded for 10 min. Climbing over or sitting on each object was excluded. The analysis of object exploration time and discrimination ratio (DR) was performed using the formula: total N time / (N time + F time) × 100, for each experimental group. The two identical objects and the arena were cleaned with 70% ethanol for each trial.

### Body weight and physiological parameter measurements

The mice were weighed weekly for the duration of the experiment and blood was collected through cardiac puncture under deep anesthesia after behavior measurements. To obtain serum, blood samples were centrifuged at 1,500 × *g* for 15 min at 4 °C. Potential electrolyte imbalance was monitored and ruled out by measuring serum concentrations of electrolytes, such as sodium (Na^+^), potassium (K^+^), and chloride (Cl^-^), using an electrolyte analyzer (Dri-Chem 3500i, Fuji, Japan).

### Sacrifice and cardiac perfusion for brain harvest

Mice abdomens were cut and cardiac perfusion was performed with PBS. In brief, the pulmonary artery was blocked, the left ventricle was pierced with a 21-gauge needle, and the needle was fixed in the ascending aorta. Immediately after starting the perfusion, the right atrium was cut with scissors. PBS and 4% paraformaldehyde (PFA) were used for perfusion and fixation. For post-fixation, the brain was soaked in 10% PFA with 10%-30% sucrose at 4 °C for 3 days, and eventually, cryosection was conducted for staining.

### Cryosection of mice brain

Mice brains were sequentially placed in 10%, 20%, and 30% sucrose solutions before freezing (in OCT compound) and then stored at -80 °C refrigerator. Thereafter, 25-μm-thick brain sections were obtained using a cryostat (Leica, Wetzlar, Germany). The sections were placed on glass slides for 12 h and then stored in -80 °C refrigerator until use.

### Analysis of pyramidal neurons in the hippocampal CA1 region using Nissl staining

Sections were dried on a slide warmer. Slides were then first immersed in an alcohol/chloroform solution overnight and then in 0.1% CV for 10 min. The slides were placed in an incubator at 40 °C, washed with distilled water one time quickly, and placed in graded ethyl alcohol (95% and 100% sequentially) for 5 min. The sections were sealed with a cover slip and mounting solution and observed under a light microscope. The density of the neuronal cell in cortical mouse brain was measured using ImageJ (NIH, MD, USA).

### Analysis of protein expression in the mouse hippocampus by western blot analysis

Mice were decapitated at predetermined time points, and the hippocampus was quickly excised and homogenized in five volumes of lysis buffer. The homogenates were centrifuged at 15,000 × *g* for 10 min at 4 °C, and the level of total protein in the supernatant was quantitated using the BSA method. Thirty micrograms of protein was separated on SDS-PAGE and transferred on a PVDF (Millipore, Darmstadt, Germany) membrane. The PVDF membranes were blocked using 5% skim milk in TBST (Tris-buffered saline, 0.1% Tween 20) for 1 h at 25 °C and incubated for 4°C overnight with primary antibodies for p-JNK (1:1000), JNK (1:1000), Mn-SOD (1:1000), and β-actin (1:1000). The membranes were then incubated with HRP-conjugated goat anti-rabbit IgG pAb (1:5000) and HRP-conjugated goat anti-mouse IgG pAb (1:3000) antibodies for 1 h at 25 °C. The membranes were then treated with the enhanced luminol-based chemiluminescent substrate (ECL) solution, and expression levels of each protein were detected using the luminescent analyzer system (Amersham Imager 600, Buckinghamshire, UK). The densities of all the detected bands were analyzed using the ImageJ program (NIH, MD, USA).

### Statistical analysis

One-way analysis of variance followed by the Holm-Sidak test was conducted to determine the statistical significance of differences among groups using the SigmaPlot 12.0 program (Systat Software Inc., CA, USA). Data are expressed as mean ± standard deviation (SD), and statistical significance was considered at *p* < 0.05.

## Results

### Effects of BCAS operation and DHST treatment on body weight change and physiological parameters in mice

**Figure 1:**
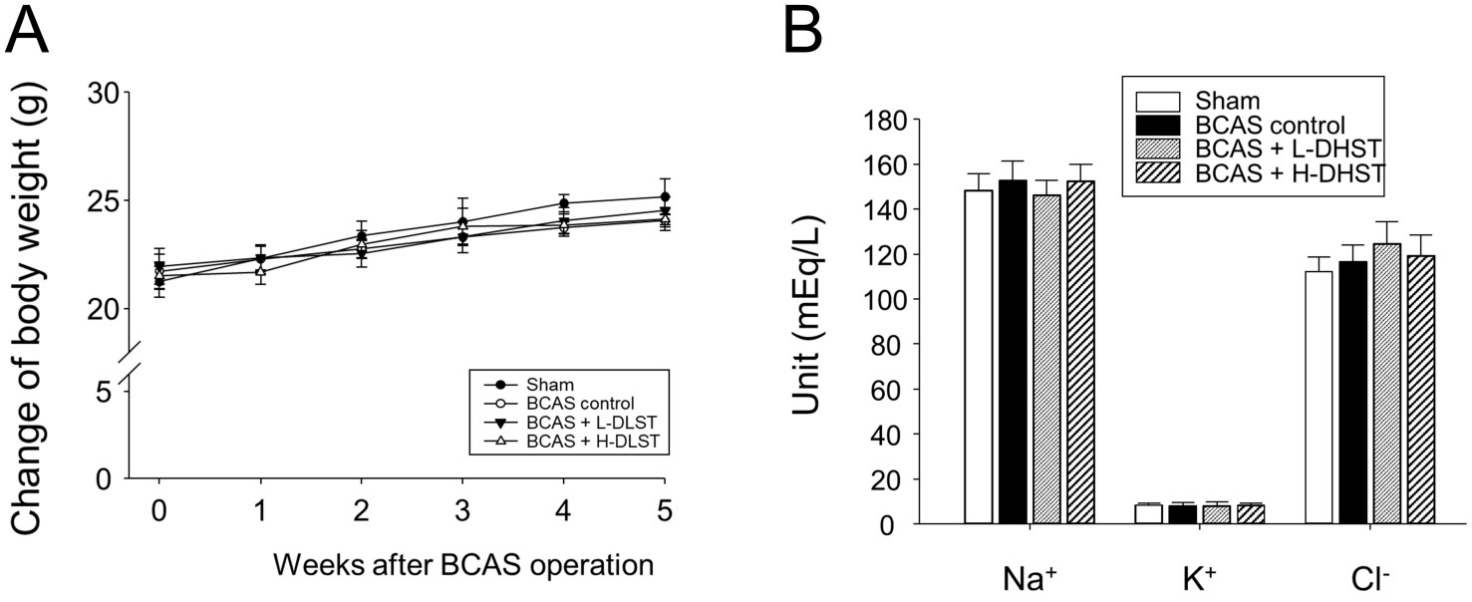
Influence of BCAS-induced VD in mice and the effect of DHST treatment on change in body weight (A) and physiological parameters (B). Mice were weighed weekly during the 5-week experimental period. Serum samples were obtained to measure the concentrations of Na^+^, K^+^, and Cl^-^. Abbreviations: BCAS, bilateral common carotid artery stenosis; DHST, Daehwang-Hwanglyoun-Sasim-Tang; VD, vascular dementia.

After BCAS operation, we observed no significant differences among the groups in physiological parameters (Na^+^, K^+^, and Cl^-^) and body weight (Figure 1A and 1B).

### Latency to first-arm entry and number of entry times into the three arms of the Y-maze

**Figure 2:**
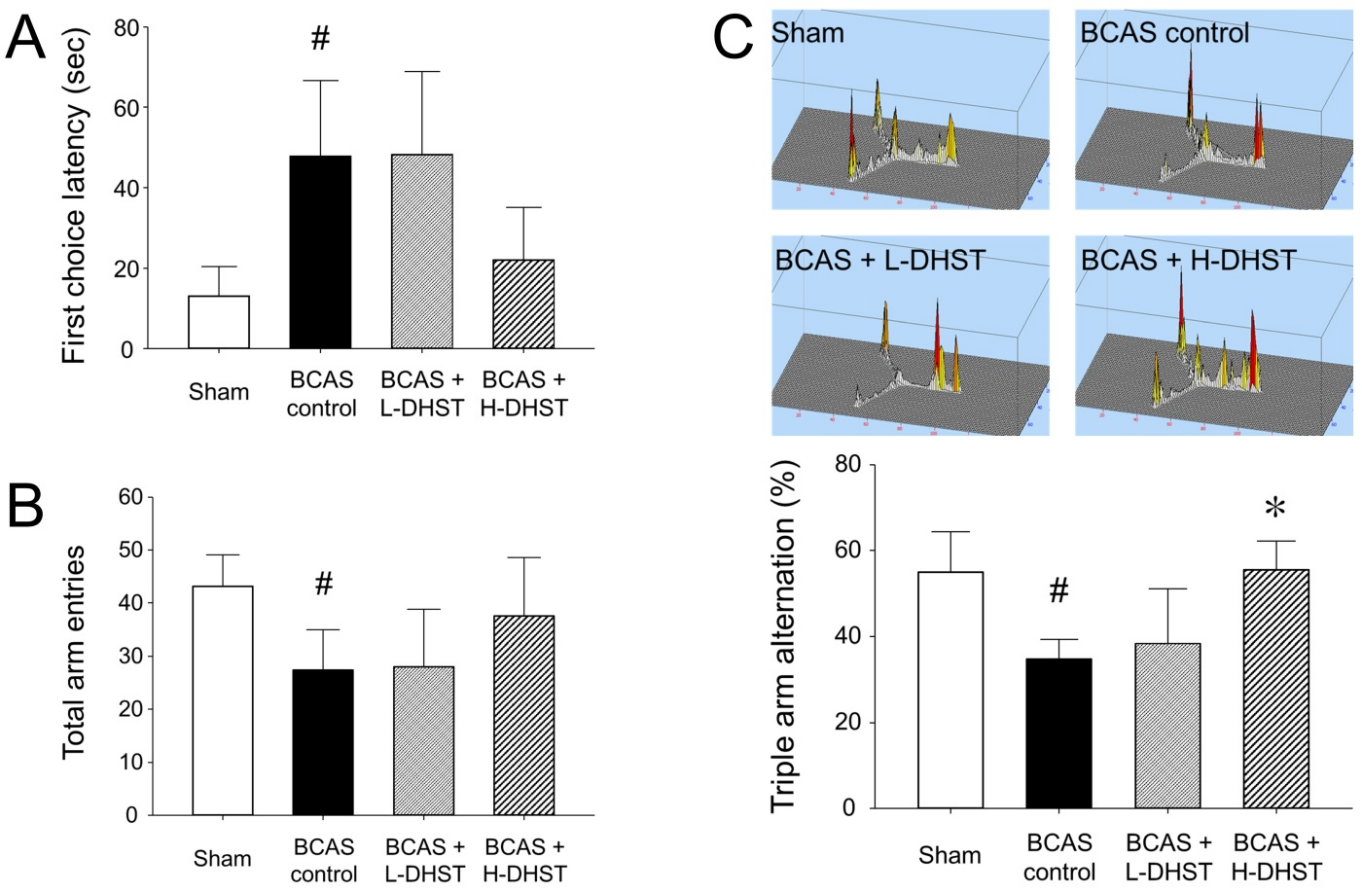
Latency to first-arm entry (A), total number of entries into the three arms (B), and probability of entering three arms sequentially (C) in the Y-maze test. DHST treatment caused no significant change on first choice latency and total arm entries; however, H-DHST treatment significantly increased triple arm alternation of BCAS-induced mice. Results are presented as mean ± SD. ^#^*p* < 0.05 vs. sham group and *p < 0.05 vs. BCAS control group; n = 5 in each group. Abbreviations: BCAS, bilateral common carotid artery stenosis; DHST, Daehwang-Hwanglyoun-Sasim-Tang.

Latency to first-arm entry in the Y-maze test was 13.07 ± 7.34 s in the sham group, 47.84 ± 18.80 s in the BCAS control group, 48.23 ± 20.74 s in the L-DHST-treated group, and 22.08 ± 12.99 s in the H-DHST-treated group (Figure 2A). The time taken for first-arm entry in the Y-maze test was significantly high in the control group. The DHST-treated groups presented no significant reduction in time taken for first-arm entry compared with the BCAS controls (Figure 2A). Figure 2B shows results for the total number of times the mice went into the three arms in the Y-maze test. The total number of times was 43.20 ± 5.89 in the sham group, 27.40 ± 7.60 in the BCAS control group, 28.00 ± 10.77 in the L-DHST-treated group, and 37.60 ± 10.99 in the H-DHST-treated group. The total number of times the mice went into the three arms in the Y-maze test was significantly lower in the control group. The DHST-treated groups presented no significant increase in the total number of times the mice went into the three arms compared with the BCAS controls.

### The probability of sequentially entering the three arms in the Y-maze

Figure 2C shows results for the probability of sequentially entering three arms in the Y-maze. The probability of triple arm alternation was 55.00% ± 9.33% in the sham group, 34.80% ± 4.48% in the BCAS control group, 38.40% ± 12.66% in the L-DHST-treated group, and 55.60% ± 6.66% in the H-DHST-treated group. The probability of sequentially entering the three arms in the Y-maze test was significantly low in the BCAS controls. The H-DHST-treated group presented a significant increase in the probability of sequentially entering the three arms compared with the BCAS controls.

### Behavioral observation results from NORT

**Figure 3:**
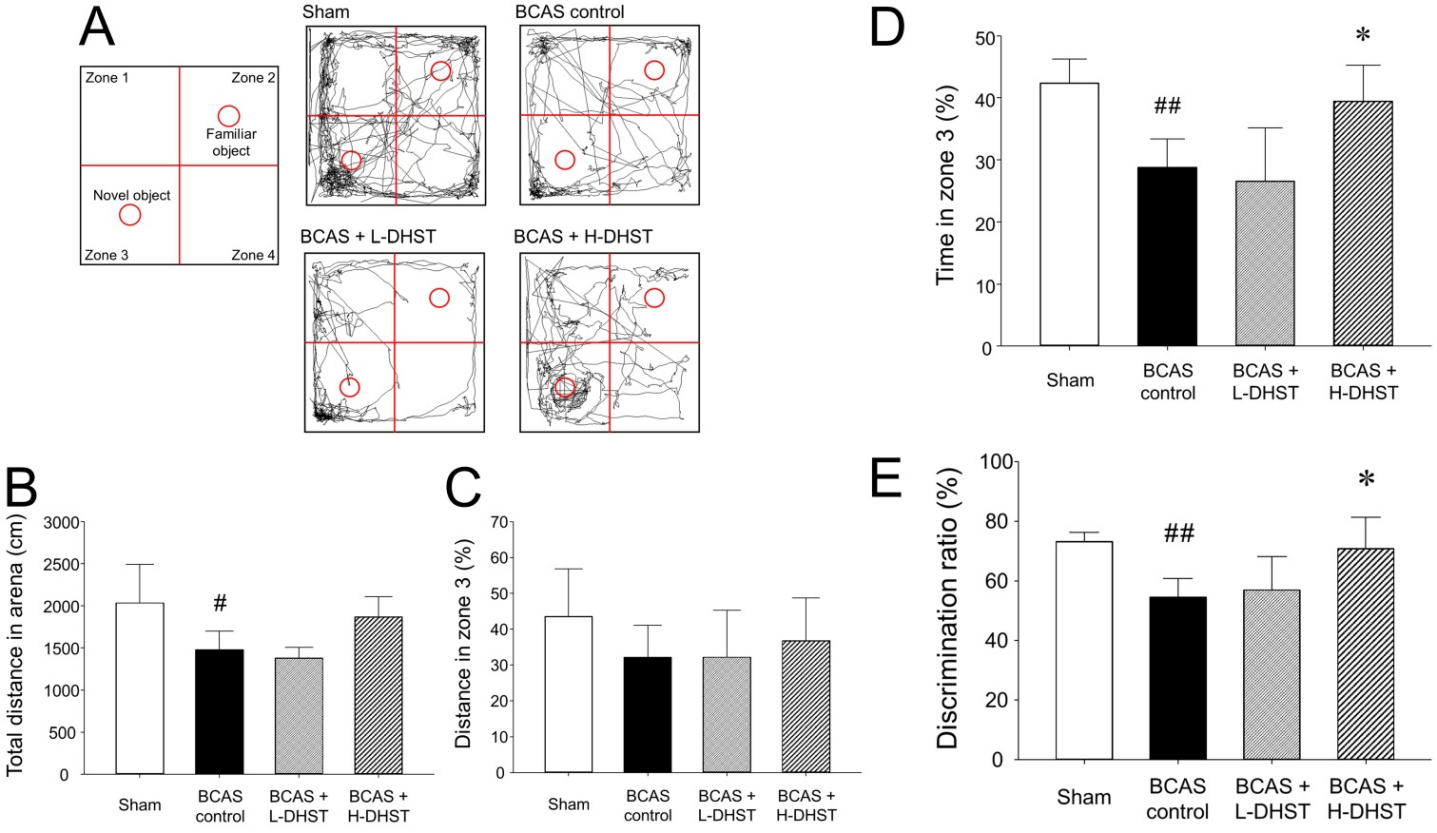
Schematic view shows NORT and the route taken (A). The total distance in the arena for NORT evaluation (B). Distance traveled in zone 3 expressed as percentage (C). The time spent in zone 3 (D) and DR (total N time / (N time + F time) × 100) (E) are expressed in percentage. H-DHST treatment significantly increased DR of BCAS-induced mice. However, DHST treatment showed no significant change in total distance covered in the arena and distance in zone 3. H-DHST treatment significantly increased time of BCAS-induced mice in zone 3. Results are presented as mean ± SD. ^#^*p* < 0.05 vs. sham group, ^##^*p* < 0.01 vs. sham group, *p < 0.05 vs. BCAS control group; n = 5 in each group. Abbreviations: BCAS, bilateral common carotid artery stenosis; DHST, Daehwang-Hwanglyoun-Sasim-Tang; DR, discrimination ratio; NORT, novel object recognition test.

NORT was used to determine if the experimental animal recognizes a new object placed in zone 3. Figure 3A shows the route taken by the mice. Figure 3B shows results for the total distance in an arena. The total distance in the arena was 1245.07 ± 1119.39 cm in the sham group, 849.52 ± 887.28 cm in the BCAS control group, 753.80 ± 882.97 cm in the L-DHST-treated group, and 1055.58 ± 1151.16 cm in the H-DHST-treated group. The total distance in the arena was significantly lower in the control group compared to the sham group. The DHST-treated groups presented no significant increase in total distance in the arena compared with the BCAS control group.

Figure 3C shows results for distance in zone 3. The distance in zone 3 (%) was 28.39% ± 21.48% in the sham group, 20.54% ± 16.39% in the BCAS control group, 22.64% ± 13.54% in the L-DHST-treated group, and 24.34% ± 17.62% in the H-DHST-treated group. When the distance traveled in zone 3 was expressed as a percentage, no significant change was observed between all groups. However, when stay-time in zone 3 was expressed as a percentage, it was significantly lower in the BCAS control group and the administration of H-DHST inhibited this change (Figure 3D). Duration time in zone 3 (%) was 42.40% ± 3.85% in the sham group, 28.80% ± 4.55% in the BCAS control group, 26.60% ± 8.56% in the L-DHST-treated group, and 39.45% ± 5.77% in the H-DHST-treated group.

DR is a measurement method frequently used for behavior analysis in NORT 33,34. DR (%) was 72.60% ± 2.88% in the sham group, 54.60% ± 6.19% in the BCAS control group, 57.00% ± 11.07% in the L-DHST-treated group, and 70.80% ± 10.45% in the H-DHST-treated group (Figure 3E).

### Morphological changes in neuronal cells in the hippocampal CA1 region

**Figure 4:**
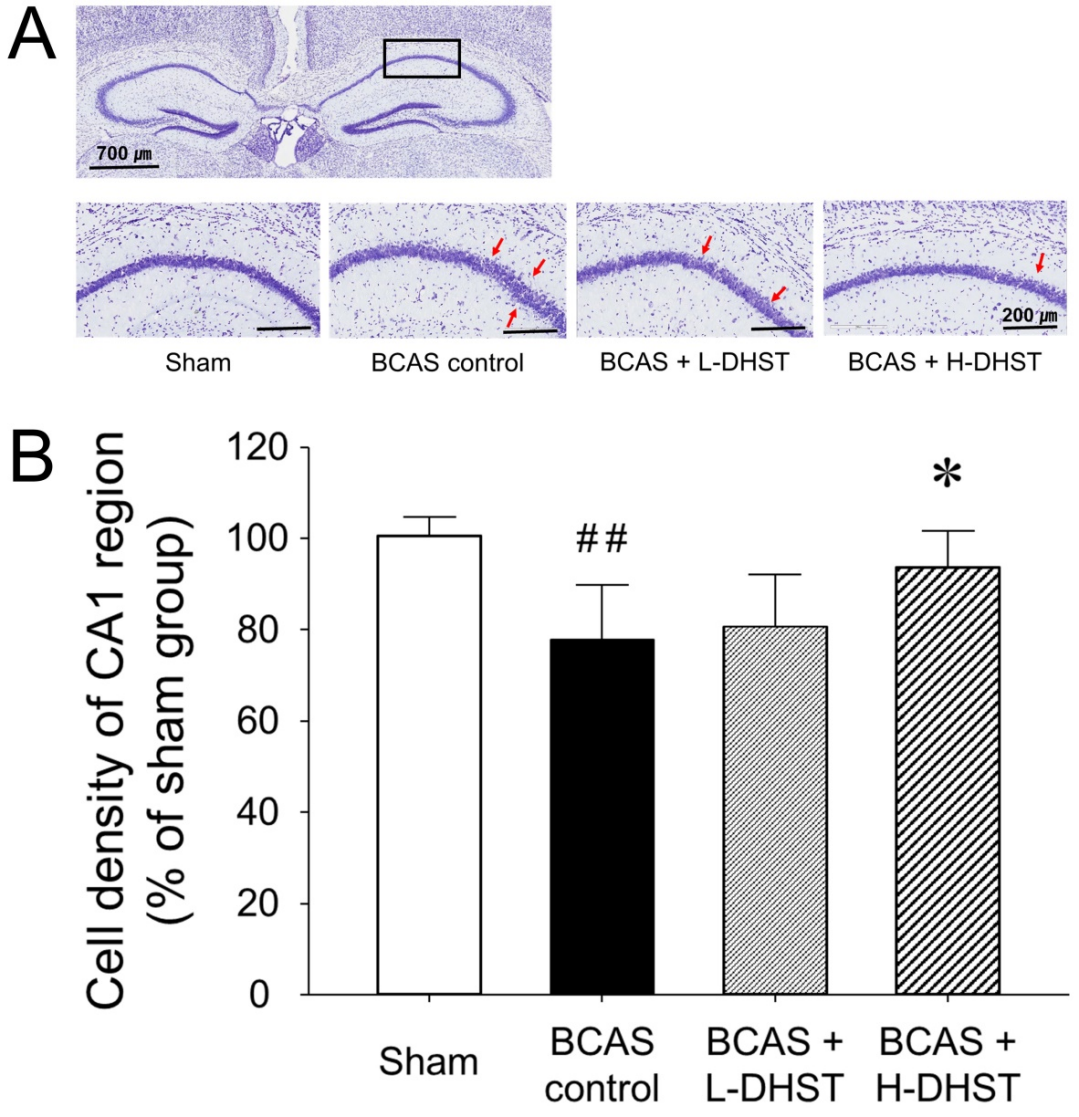
Change in morphology (A) and cell density (B) of the hippocampal CA1 region of the right hemispheric brain. Each photomicrograph of the lower column represents the CV-stained CA1 region. Red arrows indicate sparsely distributed pyramidal cell layers. Scale bars, 700 µm (upper column) or 200 µm (lower column). H-DHST treatment significantly increased the cell density of the hippocampal CA1 region in BCAS-induced mice. This diagram shows the quantitative analysis of change in cell number. Results are presented as mean ± SD. ^##^*p* < 0.01 vs. sham group, *p < 0.05 vs. BCAS control group; n = 5 in each group. Abbreviations: BCAS, bilateral common carotid artery stenosis; CV, cresyl violet; DHST, Daehwang-Hwanglyoun-Sasim-Tang.

To confirm the neuroprotective effects of DHST on BCAS-induced neuronal damage, we investigated morphological changes in neuronal cells in BCAS-induced hemispheric (right) mice brains. When chronic cerebral ischemia was maintained, pyramidal cells located in the hippocampal CA1 region were loosely distributed. In the sham group stained with CV, pyramidal cells were normal and intact, with a well-defined cytoplasm and nucleus. Color intensity did not significantly change in mouse brains treated with L-DHST. However, color intensity was significantly higher in mouse brains treated with H-DHST than in the control group (Figure 4A).

Figure 4B shows the cell density of the CA1 region upon CV staining in right hemispheric brains. The cell density was 100.65% ± 3.95% in the sham-operated group and 77.80% ± 11.92% in the BCAS control group. Moreover, the cell density was 80.80% ± 11.23% in the L-DHST-treated group and 93.60% ± 7.92% in the H-DHST-treated group. The cell density of the CA1 region was significantly lower in the control group. The cell densities of the CA1 region in mouse brains treated with L-DHST did not significantly change. However, cell density of the CA1 region was significantly higher in the H-DHST-treated group than in the control group.

### Expression of cell death-related proteins in the BCAS-induced hippocampal mouse brain

**Figure 5:**
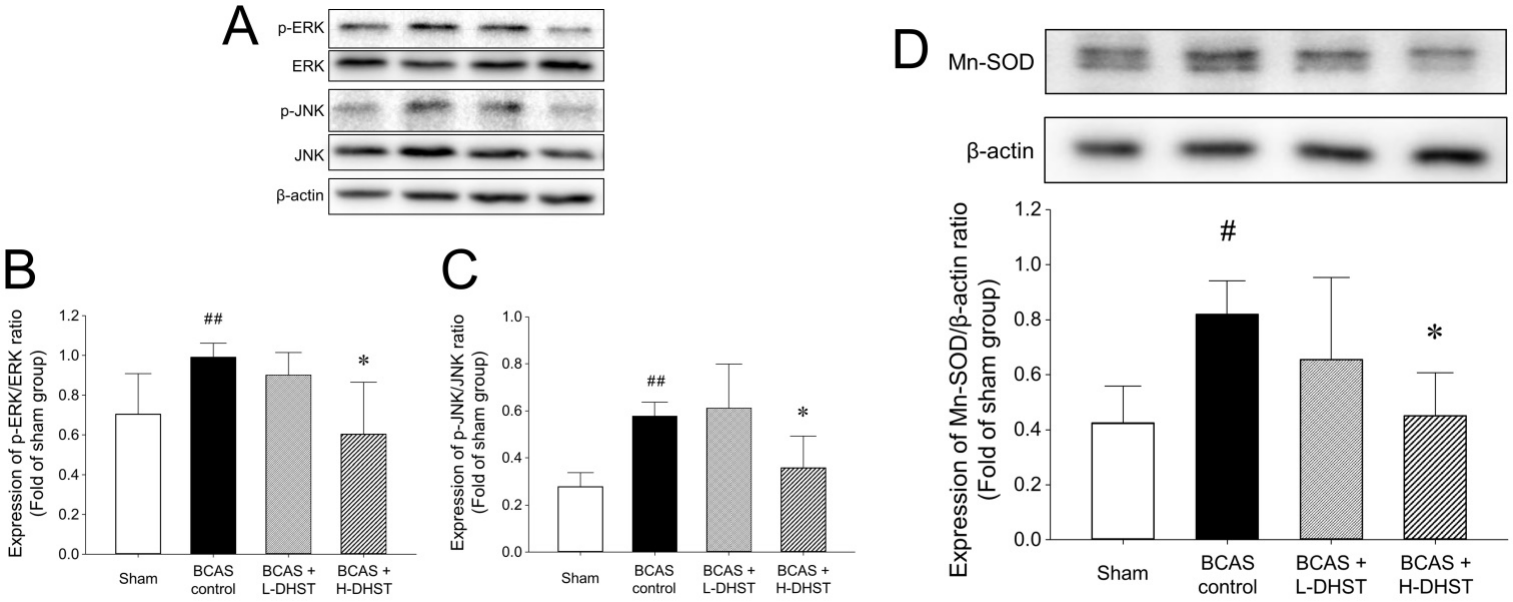
Effects of DHST treatment on changes in ERK (A and B), JNK (A and C), and Mn-SOD (D) in the bilateral common carotid artery stenosis (BCAS)-induced vascular dementia (VD) model mouse brain. Representative images and relative densitometry of western blot data for p-ERK, ERK, p-JNK, JNK, and β-actin in hippocampal brain tissues (n = 5 experiments). All data are expressed as means ± SD. ^#^*p* < 0.05 vs. sham group, ^##^*p* < 0.01 vs. sham group, *p < 0.05 vs. BCAS control group. Abbreviations: BCAS, bilateral common carotid artery stenosis; DHST, Daehwang-Hwanglyoun-Sasim-Tang; Mn-SOD, manganese superoxide dismutase.

The cellular responses to external stimuli, including growth factors, hormones, cytokine stress, and neurotransmitters, require a cascade of events that transmit signals from the cell surface to the nucleus. The mitogen-activated protein kinase (MAPK) pathway is frequently used to transduce these signals 35,36. MAPK plays a critical role in various physiologies, including cell division, differentiation, migration, and death 36. Members of the MAPK superfamily include JNK and ERK 37. Brain injury activates the MAPK pathway, causing astrocyte activation 38. Astrocyte activation is believed to depress neuronal regeneration after central nervous system injury due to the increase in proinflammatory cytokine expression, physical barrier formation, and glial scar formation 39-42. The increase in ERK expression in the BCAS control group was inhibited by H-DHST administration. The ratio of p-ERK/ERK expression was 0.70 ± 0.20 in the sham group, 0.99 ± 0.07 in the BCAS control group, and 0.60 ± 0.26 in the H-DHST-treated group (Figure 5A and 5B). The increased expression of JNK in the BCAS control group was inhibited by H-DHST administration. The ratio of p-JNK/JNK expression was 0.28 ± 0.06 in the sham group, 0.58 ± 0.06 in the BCAS control group, 0.61 ± 0.19 in the L-DHST-treated group, and 0.36 ± 0.13 in the H-DHST-treated group (Figure 5A and 5C). Mn-SOD is an important antioxidant enzyme in the mitochondrial matrix 43 and one of the first enzymes involved in ROS removal 44. Mn-SOD was reported to be associated with antioxidant pathways 45-47. Thus, Mn-SOD protects cells from oxidative damage through ROS conversion 43-47. In this study, BCAS operation significantly increased protein expression of Mn-SOD and H-DHST treatment significantly inhibited its increase. The expression values were 0.42 ± 0.13 in the sham group, 0.82 ± 0.12 in the BCAS control group, and 0.45 ± 0.16 in the H-DHST group (Figure 5D).

## Discussion

Cerebrovascular disorders reduce blood flow to the brain; hypoxia and anoxia and inflammatory responses occur sequentially. It develops in the direction of increasing oxidative stress in the brain and is accompanied by damage to the white matter and hippocampus; these pathophysiological findings ultimately appear as symptoms of VD 30. The risk of VD increases with age and approximately doubles every 5.3 years. This exponential increase is slightly less than that of Alzheimer's dementia, which doubles every 4.5 years 48. The mortality rate of VD is higher than that of Alzheimer's disease, which is thought to be due to the effect of coronary artery disease 49. According to a meta-analysis by Diniz *et al.* 50, depression that occurred later in life was found to be a risk factor for VD, similar to that in Alzheimer's disease. Changes in cognitive function in VD are much more diverse than in other diseases, such as Alzheimer's disease, because vascular lesions can occur in any part of the brain where blood vessels are distributed and depend on the affected nervous matrix 51.

There is no officially approved drug for the treatment of VD, unlike for Alzheimer's dementia, and primary treatment is limited to controlling vascular risk factors. Currently, drugs used for the treatment of VD include anticoagulants, antiplatelet agents, antioxidants, cognitive enhancers, behavioral and psychological symptom treatment for dementia, and brain metabolism promoters 2-5,9,29,30,48,50. In addition, acetylcholinesterase inhibitor and memantines are used, which have been approved for Alzheimer's disease. Evidence that VD and Alzheimer's disease share neuropathological and neurochemical factors has been the basis for the use of these drugs 52.

DHST is a commonly used prescription that consists of two herbal medicines: Rhei Rhizoma and Coptidis Rhizoma. We evaluated the anti-inflammatory and antioxidant effects of Rhei Rhizoma and Coptidis Rhizoma and proposed that DHST can be used to treat VD. To investigate their effect on cognitive function improvement, learning and memory behavior were evaluated using the Y-maze test and NORT in mice in which chronic CBF decrease was induced through BCAS. In addition, we measured the expression levels of ERK, JNK, and Mn-SOD.

One week after the BCAS operation, the DHST group was divided into high and low concentration groups. DHST was administered orally for 4 weeks to induce chronic CBF through BCAS. The results revealed that latency to first-arm entry in the Y-maze was significantly higher in the BCAS control group than in the sham group; however, the DHST-treated groups presented no significant reduction (Figure 2). Although the total number of entries into the three arms of the Y-maze was significantly lower in the BCAS control group than in the sham group, the DHST-treated groups presented no significant increase. However, the probability of entering the three arms sequentially significantly increased in the H-DHST-treated group. Behavioral evaluation using NORT revealed that the total distance covered decreased in the control group and there was no significant change in the DHST-treated group (Figure 3). When the distance covered in zone 3 was expressed as percentage, no significant change was observed between all groups, but when the time in zone 3 was expressed as percentage and DR showed a significant change in the control group, administration of a high concentration of DHST suppressed these changes.

To check the neuroprotective effects of DHST in the cortical region of hippocampal CA1, we used CV-stained frozen sections (Figure 4). The pyramidal cells of the hippocampal CA1 region are neurons that respond most sensitively to cerebral ischemia. Changes in the number of pyramidal cells and expression of various genes are the most commonly used indicators to study the mechanism of neuronal damage in cerebral ischemia and the protective effect of drugs on nerve damage 11,29,30. The cell density of the CA1 region was significantly higher in the H-DHST-treated group than in the control group. H-DHST was observed to significantly inhibit the cellular changes associated with the BCAS-induced inflammatory response.

We performed western blotting to determine the molecular mechanisms underlying the neuroprotective effects of DHST in the BCAS mouse model (Figure 5). Glutamate-induced neurotoxicity is one of the significant pathogenic mechanisms in neurological diseases such as ischemic stroke, Alzheimer's disease, and epileptic brain damage 5,29,53. Glutamate exposure was shown to induce apoptosis by increasing the levels of phosphorylated ERK and JNK 54. Our findings revealed that the expression levels of JNK and ERK increased upon BCAS induction and treatment with H-DHST reduced the levels of JNK and ERK expression (Figure 5A, 5B, and 5C). Mn-SOD has been reported as one of the important potential targets for the identification of neuroprotective agents against ischemic brain injury 43,44,46. In this study, BCAS significantly increased the expression of Mn-SOD protein, and H-DHST treatment significantly inhibited this increase (Figure 5D). This result indicates that antioxidant signaling was activated by BCAS induction and H-DHST reduced brain damage, thereby inhibiting Mn-SOD protein expression.

In summary, the H-DHST administration group exhibited improved cognitive function than L-DHST administration group. H-DHST treatment reduced neuronal cell death in the BCAS-induced mouse brain, and this effect was associated with anti-inflammatory and antiapoptotic pathways.

## Conclusions

To evaluate the effects of DHST treatment on the rodent VD model, behavioral evaluation of learning and memory was performed using the Y-maze test and NORT. In addition to evaluating the expression of apoptotic proteins, we evaluated morphological changes in the hippocampal CA1 region. The probability of the H-DHST-treated group sequentially entering the three arms of the Y-maze significantly increased compared with the BCAS control. Results from NORT analysis revealed that H-DHST treatment significantly increased the time spent in zone 3 and DR. H-DHST treatment inhibited morphological changes in pyramidal cells in the hippocampal CA1 region and suppressed the expression of JNK, ERK, and Mn-SOD in mice brains after BCAS. The cognitive function improvement effect of DHST treatment was found be associated with anti-inflammatory and antiapoptotic pathways.

## Supplementary Material

Supplementary HPLC chromatograms of Rhei Rhizoma and Coptidis Rhizoma, and their marker compounds, an overview of the experimental process, and a bird's-view of Y-maze for the spontaneous alternation test and open-field box for NORT are available in Supplementary Figures (Figure S1, S2, and S3, respectively).

## Figures and Tables

**Table 1:** The composition of Daehwang-Hwanglyoun-Sasim-Tang

Scientific name	Herbal name	Weight (g)	Ratio (%)
*Coptis japonica* Makino	Coptidis Rhizoma (黃連)	8	50
*Rheum palmatum* Linné	Rhei Radix et Rhizoma (大黃)	8	50
**Total amount**		16	100

## Abbreviations

BCAS: bilateral common carotid artery stenosis
CV: cresyl violet
DHST: Daehwang-Hwanglyoun-Sasim-Tang
DR: discrimination ratio
H-DHST: 50 mL/kg DHST
L-DHST: 5 mL/kg DHST
Mn-SOD: manganese superoxide dismutase
NORT: novel object recognition test
VD: vascular dementia
