# Supplementary material for: Effects of Daehwang-Hwanglyoun-Sasim-Tang on brain injury and cognitive function in mice caused by bilateral common carotid artery stenosis
Source: Int J Med Sci. 2022 Oct 31;19(13):1942–52. doi: 10.7150/ijms.77879 (PMC9682513; doi:10.7150/ijms.77879)
Supplement: Supplementary file 1 — Supplementary HPLC chromatograms of Rhei Rhizoma and Coptidis Rhizoma, and their marker compounds, an overview of the experimental process, and a bird's-view of Y-maze for the spontaneous alternation test and open-field box for NORT are available in Supplementary Figures (Figure S1, S2, and S3, respectively). [file ijmsv19p1942s1.pdf]

Supplementary Figure Legends

Figure S1. HPLC chromatograms of marker compounds of Rhei Rhizoma and Coptidis Rhizoma. Shimadzu HPLC system (CBM-20A, Shimadzu Technologies, Kyoto, Japan) equipped with an autosampler (SIL-20A), solvent pump (LC-20AD), oven (CTO-20A), and detector (SPD-20A) was used for analysis. Separation of the marker compounds was performed on a YMC-Triat C18 column. A and B, HPLC chromatograms of crude Rhei Rhizoma extract and its standards (sennoside A, aloe-emodin, rhein, emodin, chrysophanol, and physcion); C, HPLC chromatograms of crude Coptidis Rhizoma extract and its standard compound, berberine. A and C, 40°C column temperature, 1.0 mL/min flow rate, 10 μL injection volume; B, 32°C column temperature, 0.5 mL/min flow rate, 10 μL injection volume.

Figure S2. Overview of the experimental procedure. One week after BCAS surgery, vehicle or DHST was used for treatment five times a week for 4 weeks. Each group contained five mice. Thirty-six days after the surgery, we conducted behavior tests, which included the Y-maze test and NORT. Thirty-seven days after the surgery, the animals were euthanized and their brains were harvested for CV staining and western blotting.

Figure S3. A bird's-eye view of the Y-maze for the spontaneous alternation test (A) and open-field box for NORT (B). The Y-maze is 35 cm long, 7 cm wide, and 40 cm high. The field arena used for NORT is 40 cm long, 40 cm wide, and 40 cm high.

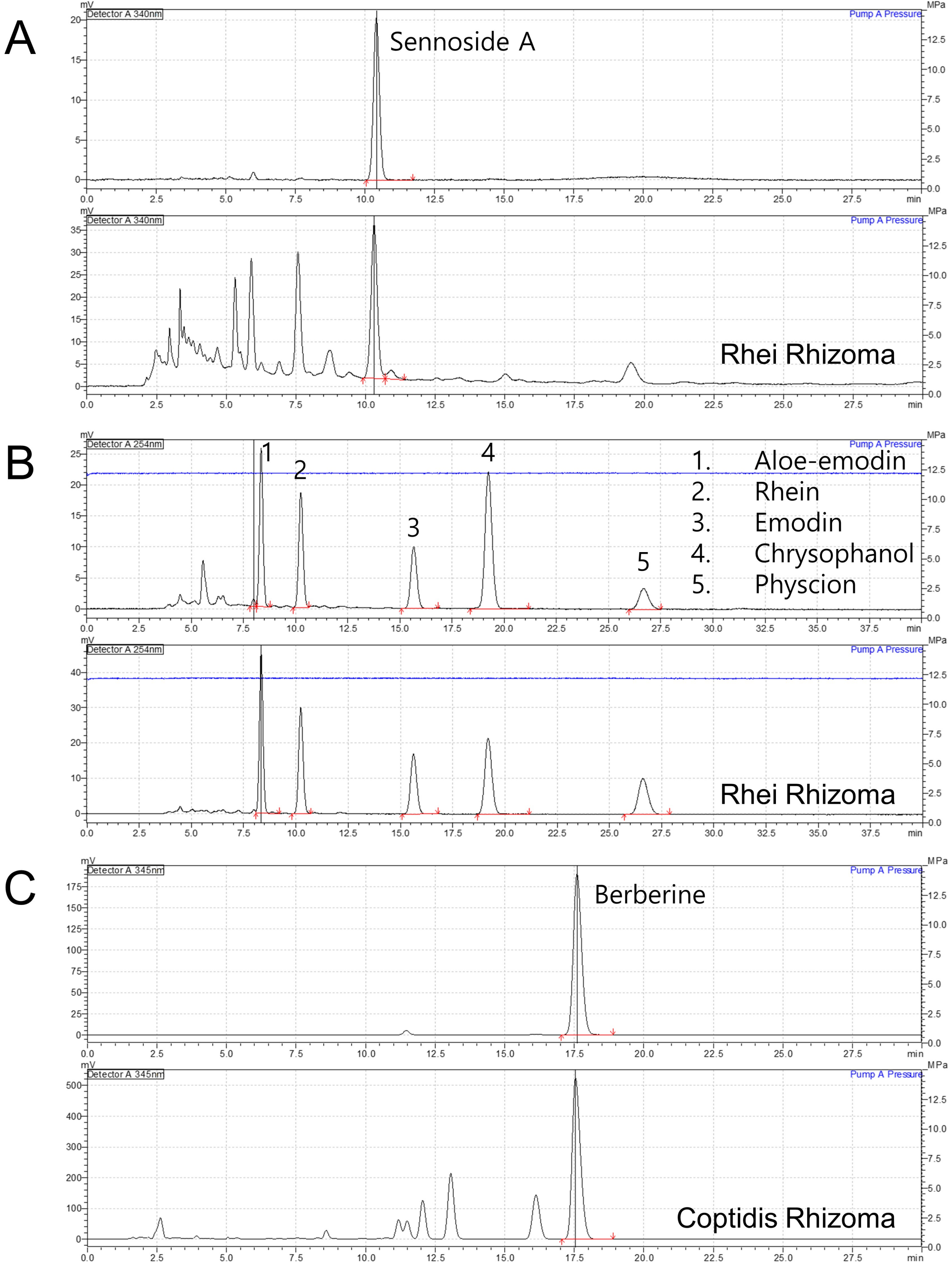

A
Sennoside A
Rhei Rhizoma
B
1. Aloe-emodin
2. Rhein
3. Emodin
4. Chrysophanol
5. Physcion
Rhei Rhizoma
C
Berberine
Coptidis Rhizoma

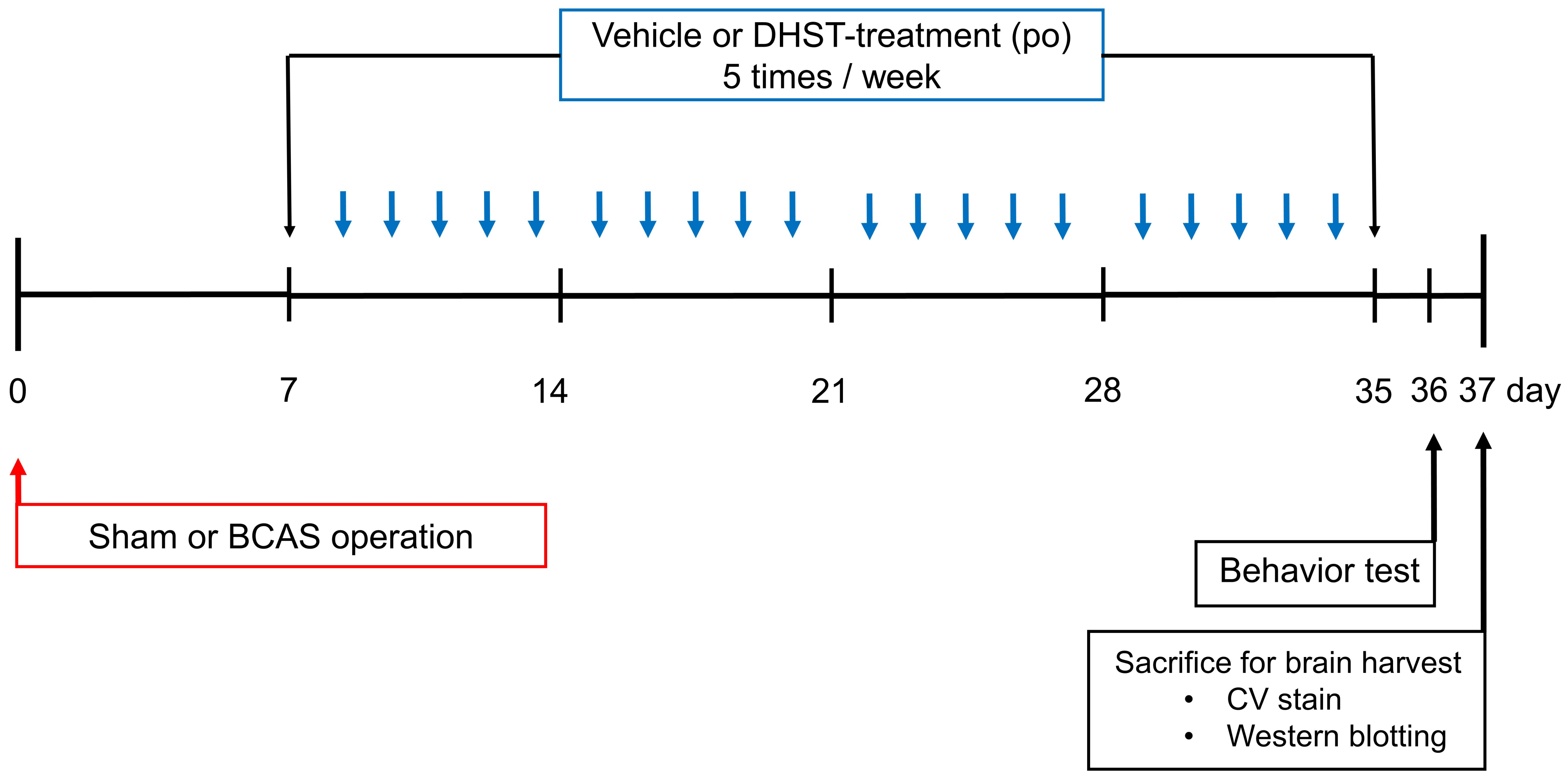

Vehicle or DHST-treatment (po)
5 times / week
0
7
14
21
28
35
36
37 day
Sham or BCAS operation
Behavior test
Sacrifice for brain harvest
• CV stain
• Western blotting

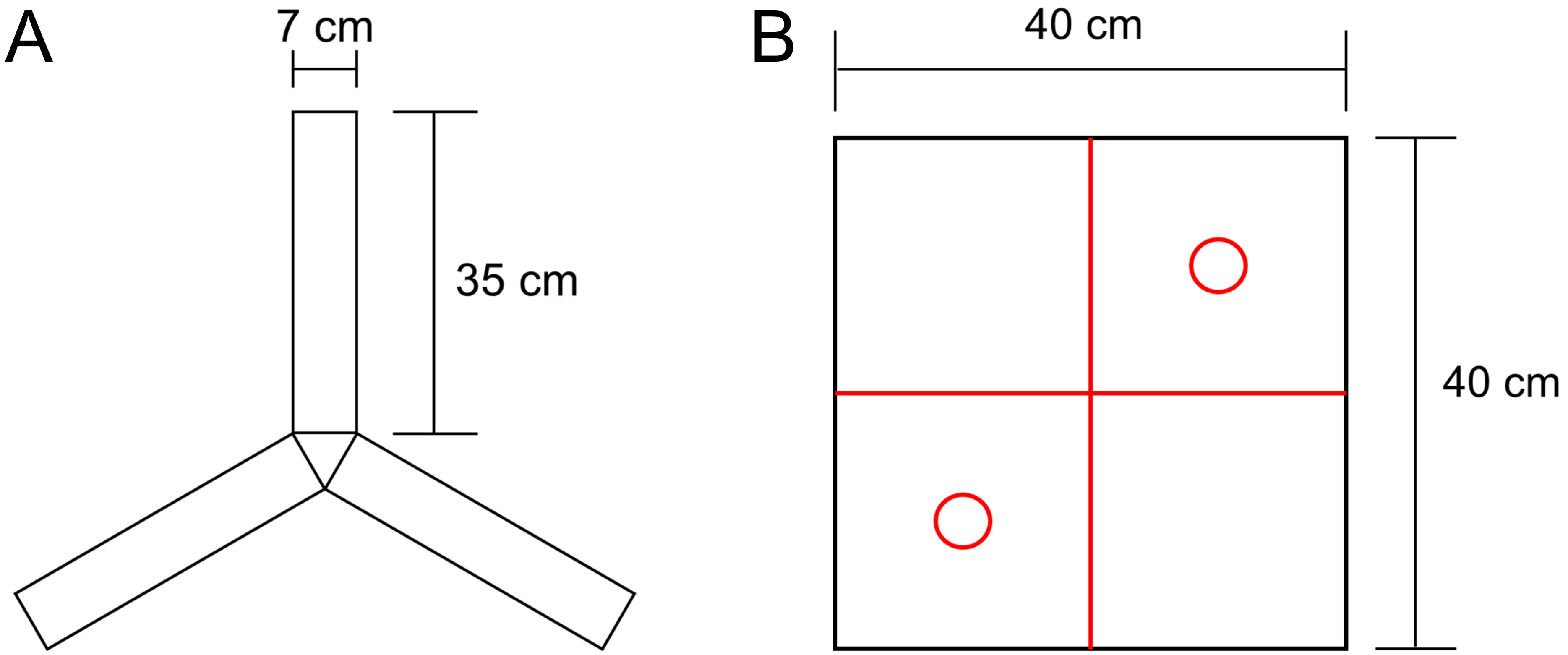
A
7 cm
35 cm
B
40 cm
40 cm
